# Rapid, Coordinate Inflammatory Responses after Experimental Febrile Status Epilepticus: Implications for Epileptogenesis

**DOI:** 10.1523/ENEURO.0034-15.2015

**Published:** 2015-11-09

**Authors:** Katelin P. Patterson, Gary P. Brennan, Megan Curran, Eli Kinney-Lang, Celine Dubé, Faisal Rashid, Catherine Ly, Andre Obenaus, Tallie Z. Baram

**Affiliations:** 1 Department of Anatomy/Neurobiology, University of California-Irvine, Irvine, California 92697; 2 Department of Pediatrics, University of California-Irvine, Irvine, California 92697; 3 Department of Neurology, University of California-Irvine, Irvine, California 92697; 4 Department of Radiation Medicine and Department of Pediatrics, Loma Linda University School of Medicine, Loma Linda, California 92350

**Keywords:** biomarker, cytokine, epilepsy, febrile seizures, heterogeneity, magnetic resonance imaging

## Abstract

Epilepsy is a common neurological disorder with many causes. For temporal lobe epilepsy, antecedent insults are typically found. These risk factors include trauma or history of long fever-associated seizures (febrile status epilepticus) in childhood. Whereas the mechanisms by which such insults promote temporal lobe epilepsy are unknown, an extensive body of work has implicated inflammation and inflammatory mediators in both human and animal models of the disorder. However, direct evidence for an epileptogenic role for inflammation is lacking. Here we capitalized on a model where only a subgroup of insult-experiencing rodents develops epilepsy. We reasoned that if inflammation was important for generating epilepsy, then early inflammation should be more prominent in individuals destined to become epileptic compared with those that will not become epileptic. In addition, the molecular and temporal profile of inflammatory mediators would provide insights into which inflammatory pathways might be involved in the disease process. We examined inflammatory profiles in hippocampus and amygdala of individual rats and correlated them with a concurrent noninvasive, amygdalar magnetic resonance imaging epilepsy-predictive marker. We found significant individual variability in the expression of several important inflammatory mediators, but not in others. Of interest, a higher expression of a subset of hippocampal and amygdalar inflammatory markers within the first few hours following an insult correlated with the epilepsy-predictive signal. These findings suggest that some components of the inflammatory gene network might contribute to the process by which insults promote the development of temporal lobe epilepsy.

## Significance Statement

Epilepsy is a devastating brain disorder affecting ∼1% of the world population. Epilepsy may arise because of genetic factors or may follow an insult or inciting event. A common antecedent of limbic, temporal-lobe epilepsy is a history of long febrile seizures in childhood. However, for unknown reasons, epilepsy develops only in some individuals who have experienced these early-life seizures. In our immature rat model of long febrile seizures, as in children, only some individuals progress to epilepsy, enabling us to address the mechanisms for these divergent outcomes. Here we find that a robust activation of some inflammatory mediators distinguishes individual rats that develop a predictive marker of epilepsy from those who do not.

## Introduction

Epilepsy is a common neurological disorder with many causes. For temporal lobe epilepsy, an adult epilepsy associated with cognitive and emotional deficits, antecedent insults are typically found. These risk factors include trauma or history of long fever-associated seizures in childhood (Cendes et al., 1993; French et al., 1993; Dubé et al., 2007).

Febrile status epilepticus (FSE) denotes long seizures (>30 min) associated with fever in early childhood (Berg and Shinnar, 1996; Berg et al., 1997). Mounting evidence suggests that FSE leads to temporal lobe epilepsy (TLE) in a subset of individuals (Annegers et al., 1987; Cendes et al., 1993; Dubé et al., 2007; Lewis et al., 2014; Seinfeld et al., 2014). Discerning the epileptogenic features of FSE has remained elusive, because in both human models (VanLandingham et al., 1998; Scott et al., 2002; Seinfeld et al., 2014) and rodent models (Toth et al., 1998, Dubé et al., 2004; Jansen et al., 2008; Hellier et al, 1998) there is evidence for neuronal stress and injury, but not for cell death, so that the epileptogenesis cannot be attributed simply to brain damage. To uncover the potential mechanisms by which FSE promotes epilepsy, several immature rodent models of FSE have been established and characterized (Baram et al., 1997; Heida et al., 2004; Dubé et al., 2010, 2000). In the model used here, ∼40% of rats exposed to early life experimental FSE (eFSE) go on to become epileptic. This fact enables a comparison of rats that become epileptic to those that do not, allowing for the dissection of processes essential for epileptogenesis (which leads to TLE) and processes that simply change during or after insult but do not contribute to the resulting epilepsy (Patterson et al., 2014).

Inflammation has been implicated in many animal models of acquired epilepsy, although its role in epileptogenesis remains unclear (Holtman et al., 2009, 2010; Vezzani et al., 2011, 2008). Fever, the initiator of FSE, intrinsically involves inflammation and the cytokine interleukin (IL)-1β is a crucial component in eFSE generation (Dubé et al., 2005). Further, higher levels of IL-1β are found in the hippocampus of animals that become epileptic (Luheshi et al., 1997; Dubé et al., 2005, 2010; Heida and Pittman,2005; Heida et al., 2009; Riazi et al., 2010; Eskilsson et al., 2014; Ravizza et al, 2008).

For these reasons, inflammation has become an attractive target for antiepileptogenic therapies (De Simoni et al., 2000; Ollikainen et al., 2004; Dedeurwerdere et al., 2012; Pouliot et al., 2013). The goal of such interventions would be to abort the epileptogenic process before spontaneous seizures begin (Vezzani et al., 2011; Patterson et al., 2014). However, several important pieces of information are lacking, impeding our ability to implicate inflammation in the processes that follow both eFSE and human FSE that may promote epilepsy. First, it is not known which of the many potential inflammatory mediators are activated by eFSE, and the time courses of their augmented expression have not been delineated. Second, it is unknown whether variations in the inflammatory response take place in individual animals that might contribute to the eventual development of epilepsy in a subset of animals.

Answering these important questions has recently been facilitated by the discovery of an early, predictive, noninvasive marker of epileptogenesis. On a high-field MRI, reduction of T2 relaxation times throughout the limbic circuit, and especially in the basolateral amygdala, within hours after eFSE predicted epilepsy in later life (Choy et al., 2014). Further, T2 signal changes correlated to an early inflammatory process in the amygdala, the translocation of high-mobility group box 1 (HMGB1). HMGB1, classically thought of as a nuclear scaffolding protein, translocates out of the nucleus during cellular stress and is then released from the cell. It acts as a proinflammatory cytokine by binding to toll-like receptor 4 (TLR4), among others, to promote an inflammatory response (Bianchi and Manfredi, 2007; Maroso et al., 2010).

Here, we probed important questions about the relationship of inflammatory cascades provoked by eFSE and their implications for epileptogenesis. Specifically, we sought to (1) delineate the temporal course of specific components of the inflammatory response in the hours and days after eFSE; (2) investigate whether and how these inflammatory mediators vary among individual animals after eFSE; and (3) correlate the variation found in inflammatory mediators in hippocampus and amygdala to the variation found in MRI signal changes. Together, these studies further our understanding of post-eFSE inflammation and how this intricate set of molecules and processes may contribute to epileptogenesis.

## Materials and Methods

### Experimental overview

To characterize the spectrum of inflammatory changes provoked by eFSE, we induced experimental seizures, then conducted quantitative mRNA analyses at several time points, buttressed by Western blot analyses and immunocytochemistry. We focused on the hippocampal formation, which is involved in both eFSE and the subsequent epileptogenesis, as well as on the amygdala, the location of the epilepsy-predicting MRI signal change. mRNA analyses were performed at multiple time points, including 1, 3, 24, and 96 h after the end of the eFSE in hippocampus, and at 24 h in amygdala. Most immunocytochemistry as well as the hematoxylin and eosin staining was performed on tissue collected 24 h after the end of the eFSE (with additional time points for HMGB1).

### Induction of experimental febrile status epilepticus

All animal procedures were performed in accordance with the regulations of the animal care committee of the University of California, Irvine. Postnatal day 10–11 Sprague-Dawley rat pups of both sexes were used for all studies. eFSE was achieved by inducing hyperthermia in pups. Pups were placed, two at a time, inside a 3 L flask, the bottom of which was lined with absorbent paper. Prior to hyperthermia, a glycerin-based hydrating ointment was applied to the paws, ears, and tail of the pups to mitigate potential hyperthermic skin injury. Pups were subjected to a continuous stream of warm air until behaviors indicating seizures began. These behaviors were identified as arrest of hyperthermia-induced hyperkinesis (freezing) followed by chewing automatisms. Core temperatures at the onset of these seizures were then rapidly measured, and seizure onset time was noted. Once seizures commenced, elevated core temperature and seizures were maintained via the warm air stream for 60 min. Seizure behaviors typically progressed over the hyperthermia period, including chewing of an extremity, clonic movements, and eventual tonic extension. The core temperature of the pups was measured every 2 min during the eFSE. If the core temperature of the pups exceeded 41.5°C, they were removed from the chamber and placed on a cool metal surface for the next 2 min. After 60 min of hyperthermia (typically 55-58 min of seizures), eFSE pups were gently and briefly immersed in cool water (∼23.0°C) to aid in the return of core temperature to normothermia and to promote seizure cessation. Pups were then placed on a euthermic pad maintained at 37°C for 30 min, and then were returned to their home cage and dam. In this study, all pups subjected to hyperthermia experienced eFSE, and there was no mortality.

### qRT-PCR Reverse transcription- PCR

Whole hippocampi or amygdalae were rapidly dissected on ice at desired time points after the end of eFSE using RNAse-free instruments. Tissue was immediately transferred to prechilled centrifuge tubes kept on dry ice. Samples were stored at −80°C until use. Total RNA was extracted from hippocampi using the mirVana miRNA Isolation Kit according to the manufacturer instructions (Ambion). Double-stranded cDNA was synthesized from total RNA using the First Strand cDNA Synthesis Kit (catalog #04379012001; Roche) with random hexamer primers. Analysis of the PCR products was executed using cDNA samples in triplicate on a LIghtcycler 96 System (Roche). Samples were normalized to β-actin and quantified using the cycle threshold method (2^ΔΔCt). Primers are provided in Table 1.

**Table 1:** Primer sequences used for RT-PCR

Figure	Data structure	Type of test	Power
2*D*; CA1 Total cells	Normal distribution	Paired *t* test	0.4974
2*D*; CA3 Total cells	Normal distribution	Paired *t* test	0.2679
2*D*; hilus Total cells	Normal distribution	Paired *t* test	0.3565
2*D*; CA1 percentage amoeboid	Normal distribution	Paired *t* test	0.0030
2*D*; CA3 percentage amoeboid	Normal distribution	Paired *t* test	0.0040
2*D*; hilus total cells	Normal distribution	Paired *t* test	0.0023
2*E*	Normal distribution	One-way ANOVA	0.0016
3*D*; CA1	Normal distribution	Paired *t* test	<0.0001
3*D*; CA3	Normal distribution	Paired *t* test	<0.0001
3*D*; hilus	Normal distribution	Paired *t* test	0.0023
3*E*	Normal distribution	One-way ANOVA	<0.0001
4*E*	Normal distribution	One-way ANOVA	0.0027
4*O*	Normal distribution	One-way ANOVA	0.6696
4*P*	Normal distribution	One-way ANOVA	<0.0001
5*A*, *B*	Normal distribution	One-way ANOVA	0.0010
5*C*	Normal distribution	One-way ANOVA	<0.0001
5*D*	Normal distribution	One-way ANOVA	0.9015
5*E*	Normal distribution	One-way ANOVA	<0.0001
5*F*	Normal distribution	One-way ANOVA	0.0064
5*H*; GFAP	Normal distribution	Paired *t* test	0.0009
5*H*; COX2	Normal distribution	Paired *t* test	0.0044
5*H*; IL-6	Normal distribution	Paired *t* test	0.4225
7*D*	Normal distribution	Linear regression	0.0016
7*E*	Non-Gaussian	Spearman *r*	0.0022
7*E*	Non-Gaussian	Spearman *r*	0.0011
7*H*	Non-Gaussian	Spearman *r*	0.0027
7*I*	Non-Gaussian	Spearman *r*	0.1541

### Western blot

Whole hippocampus from each hemisphere was dissected from rat brain 24 h post-FSE, and immediately flash frozen on dry ice and stored at −80°C until use. Tissue was homogenized in a lysis buffer containing 0.01 m Tris-HCl and 1 mm EDTA. Proteins (30 µg) were separated on either gradient gels (8-12%) or 15% polyacrylamide SDS gels (Lonza), transferred to PVDF membranes (GE Healthcare), and blocked with 5% whole milk. Membranes were then incubated with primary antibodies [GFAP 1:1000, Millipore; cyclooxygenase (Cox)-2 1:1000, Abcam; IL-6 1:2000, Abcam; and β-actin 1:20,000, Abcam] overnight at 4°C. The next day, membranes were washed in PBS-T and then incubated with HRP-conjugated secondary antibodies for 1 h at room temperature. Excess secondary antibody was removed with repeated washes in PBS-T; then membranes were visualized by repeated ECL (Pierce) exposures of various lengths and nonsaturated blots were selected, and normalized to β-actin. Western blots were analyzed using ImageJ without the knowledge of the treatment group

### Immunocytochemistry

Rats were deeply anesthetized with pentobarbital and transcardially perfused with 4% paraformaldehyde (PFA) at desired time points post-eFSE. Brains were removed and post-fixed in 4% PFA for 90 min. Brains were then cryoprotected in 30% sucrose, rapidly frozen, and stored at −80°C. Thirty micrometer sections of dorsal hippocampus were obtained on a cryostat and stored in antifreeze at 4°C until use. Serial sections were blocked in 10% normal goat serum and 0.03% Triton X in 1× PBS for 1 h at 4°C. Primary antibodies were incubated in 4% normal goat serum with 0.03% Triton X overnight at 4°C. The following antibodies were used: rabbit anti-HMGB1 1:1000 (Abcam), mouse anti-GFAP 1:3000 (Millipore), and mouse anti-IBA1 1:4000 (Wako). Sections were washed with 1× PBS, and the reaction product was visualized using 3,3'-diaminobezidine. Colocalization of cell markers with HMGB1 was achieved by coincubating rabbit anti-HMGB1 1:1000 (Abcam) with the following antibodies: mouse anti-NeuN (Chemicon), mouse anti-GFAP 1:3000 (Millipore), and mouse anti-CD11b (ABD Serotec). After 24 h of incubation, sections were washed in 1× PBS and then incubated in the appropriate secondary antibodies conjugated with Alexa Fluor 568 or Alexa Fluor 488. Colocalization was visualized using confocal microscopy.

### Hematoxylin and eosin staining

Thirty micrometer coronal sections containing dorsal hippocampus and amygdala were obtained via cryostat and dried onto glass microscope slides. Sections were rinsed two to three times in distilled H_2_O and then placed in hematoxylin solution for 5 min (Poly Scientific Research). Slides were placed under running tap water until water was no longer colored with stain and then dunked two to three times in acid alcohol (1% HCl in 70% EtOH) until sections turned pink in color. After rinsing three to five times in tap water, sections were submerged five to six times in ammonia water (1 ml of NH_4_OH in 1 L of H_2_O) to darken sections. After rinsing sections for 3-5 min in tap water, slides were submerged in Eosin Y solutions (Poly Scientific Research) for 1 min and then rinsed again in tap water. Slides were dehydrated in ethanol and xylenes, coverslipped in paramount, and allowed to dry until analysis.

### Quantification of immunocytochemistry

Quantification of activated microglia after eFSE in hippocampal CA1, CA3, and hilus was achieved by boxing off 1000 × 500 μm areas of each studied region. First, all IBA1^+^ cells in the defined region were counted and compared among groups. Activated microglia were identified as IBA1^+^ cells that had large, dark soma and few short processes that were thicker in appearance than nonactivated microglia (Lenz et al., 2013). Microglia activation is presented as the percentage change in activated microglia over the total number of IBA^+^ cells. Quantification of GFAP^+^ cells was achieved by using the same hippocampal regions and area delineations. GFAP^+^ cells were counted and compared among groups. HMGB1 quantification was accomplished by counting all HMGB1^+^ cells in a 100 × 500 μm region of CA1. Translocation of HMGB1 protein was identified by the presence of immunoreactivity outside the nucleus (i.e., in the somatic cytoplasm and in the processes of cells). Change in HMGB1 translocation is presented as the percentage change in translocated cells over the total number of HMGB1^+^ cells.

### MRI procedure and analysis

MRIs used a Bruker Avance 11.7 T MR scanner. Rats were anesthetized with 1.5% isoflurane in 100% O_2_ and remained anesthetized for the duration of the scan. Their body temperature was maintained at ∼36.5°C via a warm-water cushion underneath them. A field-of-view of 2.3 cm and a slice thickness of 0.75 mm were used for scans. T2-weighted images were acquired using a 2D multi-echo-spin-echo sequence with the following parameters: TR, 4697 ms; TE, 10.21–100.1 ms; inter-TE, 10.21 ms; matrix size, 192 × 192; and number of averages, 2. Animals were imaged 6 h after the end of eFSE. Control and eFSE animals were intermixed to maximize the homogeneity of the imaging parameters. Images were coded and analyzed without knowledge of treatment group. Basal amygdalae were delineated manually and separately in the left and right hemispheres. T2 values were measured using ImageJ software in which the images were defined on grayscale maps and a dynamic range of 1–100 ms was used. MRI signal changes are frequently unilateral both in children with FSE and in rodent models of FSE (Dubé et al., 2009b; Choy et al., 2014). Therefore, each amygdala was analyzed separately, and the side with the lower T2 relaxation time was used. The same approach was taken for the control rats, so that the amygdala with the lower T2 value was compared in all groups.

## Results

### Description and parameters of the inciting eFSE

**Figure 1.**
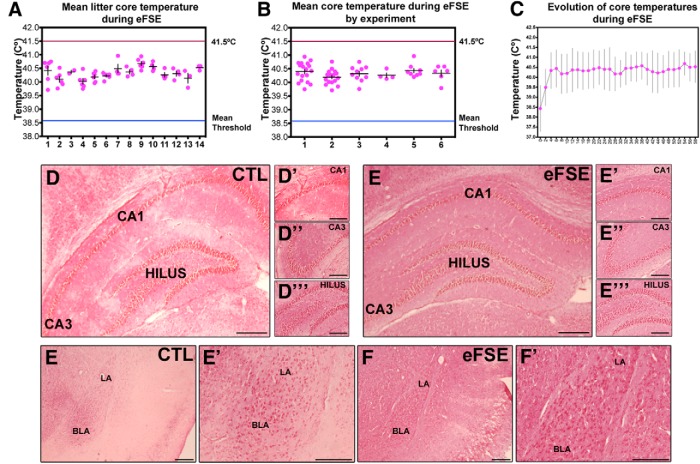
eFSE parameters. ***A***, No statistical differences of mean core temperature exists between litters (or day of eFSE induction; *n* = 61 over 14 litters). ***B***, eFSE data arranged by experimental group show no difference in mean core temperatures during eFSE (*n* = 61 experimental groups are distinguished with numbers 1–6 on the *x*-axis). No animal used in this study had a mean core temperature during eFSE that exceeded 41.5°C. ***C***, Elevated core temperatures after the first 2 min of eFSE were similar for the duration of eFSE (*n* = 61 time of core temperature reading are delineated in 2 min epochs across the *x*-axis). No overt differences in structure or cell fallout were apparent 24 h after the end of eFSE in hippocampus or amygdala using H&E staining (***D–F'***). CTL, Control. LA, lateral amygdala. BLA, basolateral amygdala.

All animals exposed to hyperthermia developed eFSE. Mean core temperatures did not significantly differ between litters or the experimental groups during 60 min of sustained eFSE (Fig. 1*A*,*B*). Further, mean core temperatures did not rise above 41.5°C in any animal, nor did they drop below the mean seizure threshold. In general, once seizures commenced (average, 3.9 min; Dubé et al., 2007), core temperatures did not significantly change during the course of hyperthermia (Fig. 1*C*). Twenty-four hours after the end of eFSE, hematoxylin and eosin staining did not suggest overt neuronal staining or dropout in dorsal hippocampus or amygdala from rats experiencing eFSE (Fig. 1*D–F'*). These nonquantitative observations are in line with previous exhaustive quantitative analyses in several rat cohorts subjected to the same model and following the same duration of hyperthermia (Toth et al., 1998; Dubé et al., 2010)

### Activation of astrocytes and microglia in hippocampus after FSE

**Figure 2.**
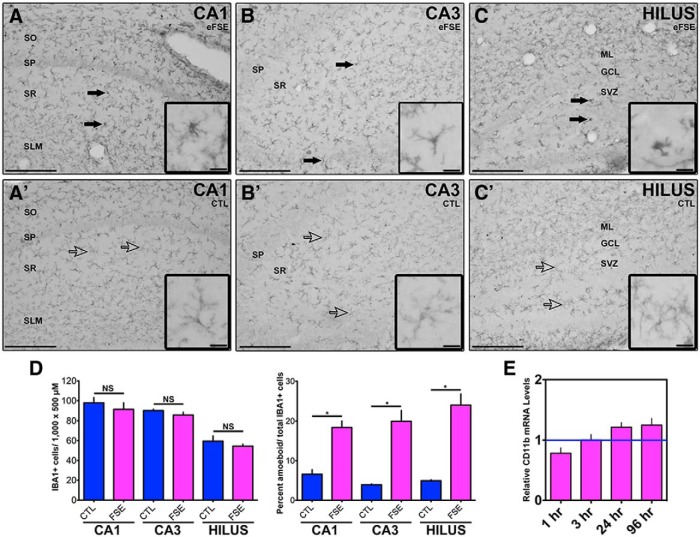
Activated microglia after eFSE. IBA1-IR cells are visible 24 h after eFSE in dorsal hippocampus in both eFSE rats (***A***, ***B***, ***C***) and control rats (***A'***, ***B'***, ***C'***). ***D***, While the number of IBA1-IR cells is similar between the eFSE group (*n* = 4) and control group (*n* = 4), eFSE rats have a larger percentage of amoeboid microglia. Ramified (or nonactivated) microglia have slender, long processes with small soma, while amoeboid (or activated) microglia have short, thick processes with a distinct large soma. Solid arrows point to amoeboid microglia, while empty arrows point to ramified microglia. ***E***, There are no differences in mRNA levels for CD11b (1 h, *n* = 5; 3 h, *n* = 6; 24 h, *n* = 6; 96 h, *n* = 4), which can increase as microglia become active. Data are presented as the mean ± SEM. Scale bars: ***A–C'***, 200 μm; insets, 5 μm. *Statistically significant at *p* < 0.05. CTL, Control. SO, stratum oriens. SP, stratum pyramidale. SR, stratum radiatum. SLM, lacunosum- moleculare. ML, molecular layer. GCL, granule cell layer. SVZ, subventricular zone.

The number of cells expressing the microglia marker IBA1 (Fig. 2*A–C'*) was not different between the control and eFSE groups 24 h after the insult. However, there were changes in the shape of IBA1-immunoreactive (IR) cells between groups (Fig. 2*D*). In the control group, IBA1-IR cells had long processes, while many cells in the eFSE group had no processes or were more globular in shape (Fig. 2*A–C'*). IBA1-IR cells with long processes are regarded as ramified microglia (nonactivated), indicative of a quiescent state. Globular IBA1-IR cells with short or nonvisible processes are indicative of activated or amoeboid microglia (Lenz et al., 2013). Because activated microglia produce CD11b, we measured CD11b mRNA levels. There were no significant changes in the expression of this molecule in the post-eFSE hippocampus at any time point (Fig. 2*E*).

**Figure 3.**
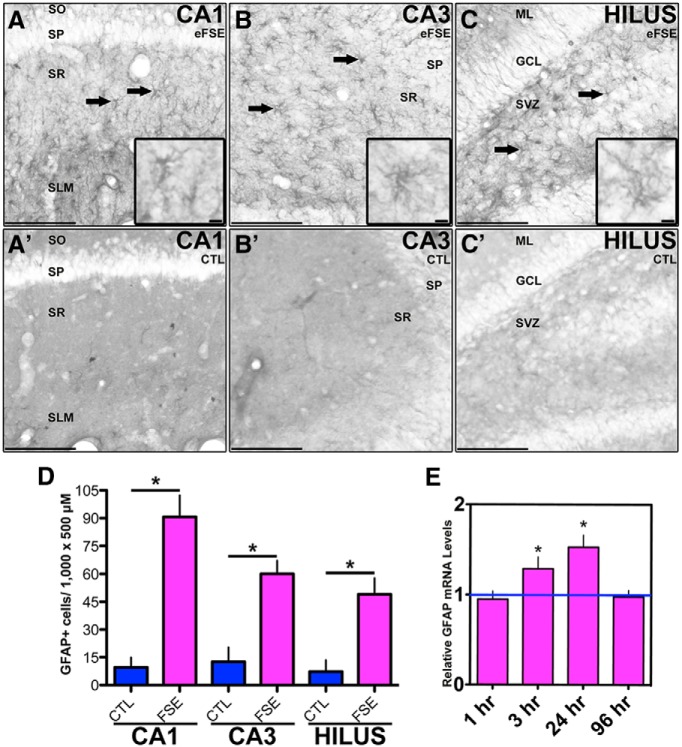
Activated astrocytes after eFSE. GFAP-IR cells are visible in dorsal hippocampus regions CA1, CA3, and the hilus of the dentate gyrus 24 h after eFSE (***A***, ***B***, ***C***; *n* = 4) but not in control rats (***A'***, ***B'***, ***C'***; *n* = 4). Solid arrows depict GFAP^+^ cells. ***D***, Quantification reveals a significant increase in the number of GFAP-IR cells compared with controls. ***E***, mRNA levels of GFAP are significantly increased 3 and 24 h after eFSE, but not at 1 or 96 h (1 h, *n* = 5; 3 h, *n* = 6; 24 h, *n* = 6; 96 h, *n* = 4). Data are presented as the mean ± SEM. Scale bars: ***A–C'***, 200 μm; insets, 5 μm. *Statistically significant at *p* < 0.05. CTL, Control. SO, stratum oriens. SP, stratum pyramidale. SR, stratum radiatum. SLM, lacunosum- moleculare. ML, molecular layer. GCL, granule cell layer. SVZ, subventricular zone.

In hippocampi from rats killed 24 h following eFSE, the number of GFAP-IR cells increased compared with controls in hippocampal CA1, CA3, and the hilus of dentate gyrus (Fig. 3*A–D*). GFAP mRNA levels were increased at 3 and 24 h after eFSE, and returned to baseline levels at 96 h, as measured with RT-PCR reverse transcription- PCR (Fig. 3*E*). Together, these data indicate that GFAP production was increased in astrocytes after eFSE, which is consistent with the activation of this cell population.

### HMGB1 translocation in hippocampal neurons after eFSE

**Figure 4.**
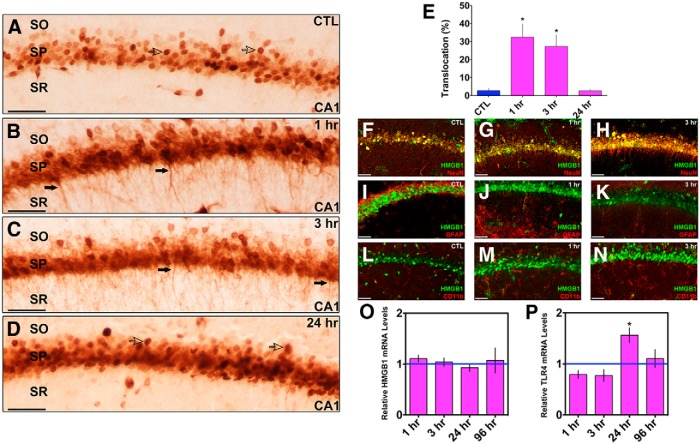
HMGB1 translocation in hippocampal neurons after eFSE. ***A***, In control animals (*n* = 3), HMGB1 is confined to the nucleus of cells (open arrows) in area CA1 of dorsal hippocampus. ***B***, One hour after the end of eFSE, HMGB1-IR is seen in the processes of neurons (closed arrows; *n* = 4), indicating translocation of HMGB1 from the nucleus. ***C***, Translocation is still evident 3 h after the end of eFSE (*n* = 4). ***D***, By 24 h after eFSE, mostly nuclear HMGB1 is observed (*n* = 4). ***E***, Quantification of HMGB1 immunocytochemistry as the percentage of HMGB1-IR cells with translocation over total HMGB1-IR cells shows a significant increase in the percentage of HMGB1 translocation 1 and 3 h after eFSE with a return to control conditions by 24 h. ***F–M***, Double labeling of HMGB1 and NeuN (both in soma and processes) demonstrates that HMGB1 translocation occurs from neurons (***F–H***) and not from astrocytes, as shown with GFAP (***I–K***), or microglia, as shown with CD11b (***L***, ***M***; CTL *n* = 3; 1 h, *n* = 4; 3 h, *n* = 4). ***O***, There is no increase in the levels of HMGB1 mRNA, indicating that translocation is achieved using already available protein (1 h, *n* = 5; 3 h, *n* = 6; 24 h, *n* = 6; 96 h, *n* = 4). ***P***, TLR4, one receptor through which proinflammatory HMGB1 acts, mRNA is upregulated 24 h after eFSE with a return to baseline values by 96 h (1 h, *n* = 5; 3 h, *n* = 6; 24 h, *n* = 6; 96 h, *n* = 4). Data are presented as the mean ± SEM. Scale bars, 100 μm. *Statistically significant at *p* < 0.05. CTL, Control. SO, stratum oriens. SP, stratum pyramidale. SR, stratum radiatum.

Our previous studies demonstrated HMGB1 translocation from nuclei to cytoplasms of amygdala neurons in parallel with the appearance of T2 signal changes in the amygdala of the same rats. Here we focused on hippocampus, the origin of spontaneous seizures in rats that become epileptic after eFSE. HMGB1 was localized to the nuclei of cells in control CA1 neurons (Fig. 4*A*). At 1 and 3 h after eFSE, HMGB1-IR appeared in the cytoplasm, which is indicative of translocation of this protein (Fig. 4*B*,*C*). Cytoplasmic HMGB1 was no longer present 24 h after eFSE (Fig. 4*E*). HMGB1 colocalized with NeuN-IR hippocampal cells, but not with GFAP-IR or CD11b-IR cells (Fig. 4*F–N*). No changes in hippocampal HMGB1 mRNA levels were found at any of the examined time points (Fig. 4*O*). mRNA expression of one of the target molecules of HMGB1, TLR4, significantly increased at 24 h after eFSE (Fig. 4*P*) and was back at baseline levels 4 d after the eFSE. Together, these data suggest a rapid yet transient translocation of hippocampal HMGB1 from the nucleus to the cytoplasm, likely followed by secretion from the cell and activation of target receptors. This process took place in hippocampal neurons, but not in microglia or astrocytes.

### Cytokine expression after eFSE

**Figure 5.**
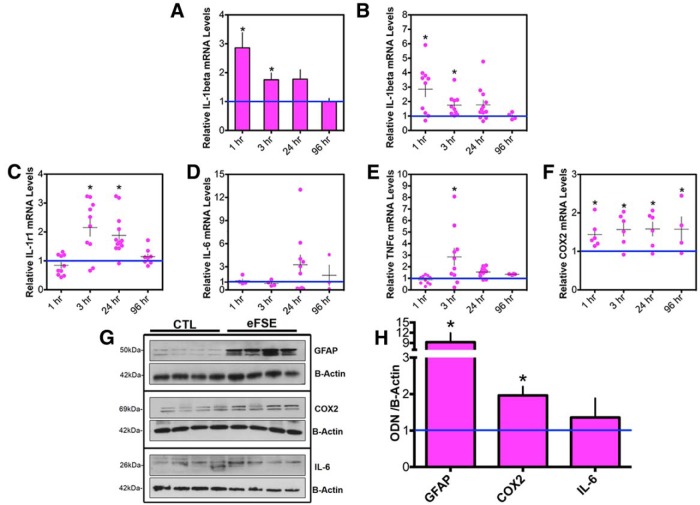
Transient cytokine upregulation after eFSE. ***A***, IL-1β mRNA levels are significantly upregulated 1 and 3 h after eFSE (CTL = 23; 1 h, *n* = 10; 3 h, *n* = 10; 24 h, *n* = 12; 96 h, *n* = 4). ***B***, Variability of IL-1β mRNA expression between rats is apparent when data are presented as individual animals where some rats have high levels of IL-1β expression and others are indistinguishable from controls. ***C***, IL-1r1 mRNA levels are significantly upregulated 3 and 24 h after eFSE, with obvious interanimal variability (CTL, *n* = 20l; 1 h, *n* = 10; 3 h, *n* = 10; 24 h, *n* = 13; 96 h, *n* = 4). ***D***, While never statistically significant, IL-6 levels trend upward for some animals at 24 h with a return to baseline values by 96 h (CTL, *n* = 9; 1 h, *n* = 4; 3 h, *n* = 4; 24 h, *n* = 10; 96 h, *n* = 4). ***E***, TNF-α mRNA levels are significantly augmented by 3 h and return to baseline by 24 h (CTL, *n* = 25; 1 h, *n* = 10; 3 h, *n* = 10; 24 h, *n* = 12; 96 h, *n* = 4). ***F***, COX2 mRNA levels are significantly upregulated at every tested time point (CTL, *n* = 11; 1 h, *n* = 6; 3 h, *n* = 6; 24 h, *n* = 6; 96 h, *n* = 4). ***G***, GFAP, COX2, and IL-6 protein levels are augmented with interanimal variation 24 h after eFSE (*n* = 4) compared with littermate controls (*n* = 4). Densitometric analyses of protein levels normalized and compared with controls are provided in ***H***. Data are presented as the mean ± SEM. *Statistically significant at *p* < 0.05.

IL-1β mRNA levels increased significantly in hippocampus 1 and 3 h after eFSE. Levels then declined, so that, by 96 h after eFSE, IL-1β mRNA levels were indistinguishable from those of control rats (Fig. 5*A*). Notably, there was a significant interanimal variability in the levels and temporal kinetics of IL-1β mRNA expression (Fig. 5*B*).

Interanimal variability was prominent upon examination of the time courses of expression of all other investigated cytokines. Levels of IL-1r1 mRNA (the IL-1β receptor) increased significantly 3 and 24 h after eFSE, and returned to baseline levels by 96 h (Fig. 5*C*). No augmentation of IL-6 mRNA was found at 1 and 3 h after eFSE, but levels were increased by 24 h in a subset of rats and returned to baseline in all rats by 96 h (Fig. 5*D*). Hippocampal TNF-α mRNA was significantly augmented 3 h after eFSE and returned to baseline by 24 h (Fig. 5*E*). COX2 expression was significantly enhanced at all tested time points after eFSE and remained upregulated at 96 h, the last time point assessed (Fig. 5*F*).

To examine whether augmented mRNA expression of cytokines translated into increased levels of functional inflammatory mediators, protein levels of GFAP, COX2, and IL-6 were measured in hippocampi 24 h after eFSE (Fig. 5*G*,*H*). These specific proteins were chosen because of the upregulation of their mRNA 24 h after eFSE in some but not all rats. We found a significant increase in protein levels of COX2 and GFAP in hippocampus, with interanimal variability, which were similar to the findings at the mRNA level. Similar to mRNA levels, protein levels of IL-6, measured in hippocampus 24 h after eFSE, were not consistently or significantly increased.

### Distinct inflammatory cytokine profiles in individual rats after eFSE

Following eFSE, ∼40% of rats develop epilepsy (Dubé et al., 2010; Choy et al., 2014). If inflammatory molecule activation contributes to epileptogenesis after eFSE, the prediction is that there will be strong individual variation in cytokine expression, with robust changes in ∼40% of rodents experiencing eFSE. We tested this possibility by delineating the inflammatory cytokine profile of individual rats.

**Figure 6.**
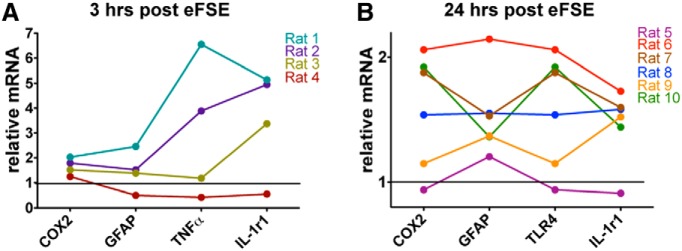
Interanimal variation of inflammatory mediator expression after eFSE. ***A***, Three hours after eFSE, COX2, GFAP, TNF-α, and IL-1r1 levels were significantly increased. When the expression profile of an individual rat is examined across these mediators, trends in expression appear (*n* = 4). For example, rat 1 has the highest expression across all explored mediators, while rat 4 has virtually no inflammatory response after eFSE. Rats 2 and 3 both fall in between rats 1 and 4, with moderate expression profiles. ***B***, A similar pattern is found 24 h after eFSE as well (*n* = 6). COX2, IL-1r1, TLR4, and GFAP were all significantly upregulated at this time point. As seen with the 3 h time point, rats with high expression in one mediator have high expression in all others, and vice versa. Rat 6 has the highest expression of all examined mediators, while Rat 5 is a nonresponder.

The expression of different cytokines peaks at different time points; therefore, the time point for the maximal expression of each cytokine was included in the composite molecular profile of each rat experiencing eFSE (Fig. 6). By 3 h after the end of eFSE, mRNA levels of COX2, GFAP, TNF-α, IL-1β, and IL-1r1 were significantly augmented (Fig. 6*A*). In individual animals, the expression levels of these cytokines tended to covary: animals with high expression of one cytokine tended to have higher expression of the others, whereas animals with low expression of one cytokine had depressed levels of the others. This trend was also found at the 24 h time point, when IL-1r1, COX2, and TLR4 were significantly upregulated in a subset of rats (Fig. 6*B*).

### Expression levels in hippocampus and amygdala of a subset of inflammatory mediators correlate with a predictive marker of post-eFSE epilepsy

**Figure 7.**
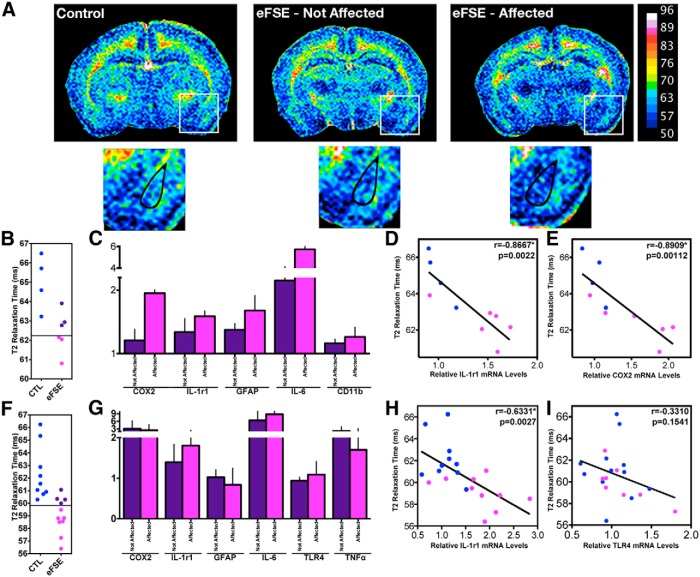
Inflammation profiles of individual animals correlate with predictive measures of post-eFSE epilepsy. *A*, Representative color-coded quantitative T2 maps 4–6 h after the end of eFSE from a control animal (*n* = 9), an eFSE animal not expected to become epileptic (eFSE-Not Affected; *n* = 5), and an eFSE animal expected to become epileptic (eFSE-Affected; *n* = 7). Basolateral amygdala (whose T2 relaxation time is used to predict epilepsy) is highlighted in enlarged pictures below. Cooler colors (blues) represent faster T2 relaxation times, while warmer colors (greens and oranges) indicate slower T2 relaxation times. ***B***, Prediction of epilepsy after eFSE is determined when T2 relaxation times of eFSE fall 2 SDs below the control mean, represented here by a black line across the *y*-axis. ***C***, In hippocampus, the expression of inflammatory molecules is higher in animals expected to become epileptic (Affected) compared with those not expected to become epileptic (Not Affected). ***D***, ***E***, Inverse correlations are found between T2 relaxation times and IL-1r1 (***D***) and COX2 (***E***; CTL, *n* = 4, eFSE *n* = 6). ***F***, For cytokine correlations in amygdala, 7 of 11 eFSE animals had T2 values low enough to consider them affected. ***G***, Cytokine mRNA data from amygdalar punches 24 h after the end of eFSE demonstrate lower discrimination between affected and nonaffected animals. ***H***, ***I***, Correlations between predictive MRI signal changes were present with IL-1r1 mRNA (***H***), and a weak correlation existed between T2 levels and TLR4 (***I***; CTL, *n* = 9; eFSE, *n* = 12). Data are presented as the mean ± SEM. *Statistically significant at *p* < 0.05. CTL, Control.

We previously investigated potential noninvasive predictive markers for the epileptogenesis that, in a subset of rats, follows eFSE. We found that decreased MRI T2 relaxation times in the amygdala 2-4 h after eFSE predicted whether or not an individual animal developed spontaneous seizures that involved, and seem to originate from, the hippocampus (Choy et al., 2014). Here we capitalized on the epilepsy-predicting MRI signal change, and studied whether augmented expression of hippocampal cytokines in individual rats correlated in the same animal with reduced T2 values. In this cohort, MRI T2 relaxation time was reduced in a subset of eFSE rats 6 h after insult compared with controls (Fig. 7*A*). Three of six eFSE rats in this cohort had T2 relaxation times that were lower than 2 SDs from the control mean, the cutoff for considering the signal abnormal and predictive of epilepsy in later life (Fig. 7*B*). We assessed hippocampal cytokine expression in the same rats 24 h after eFSE. The expression of a number of inflammatory mediators tended to be higher in the rats in which MRI signal changes were found (Fig. 7*C*). In addition, the expression of a number of individual inflammatory mediators correlated significantly and inversely with T2 relaxation times. This correlation was largely limited to inflammatory molecules that were upregulated in hippocampus 24 h after the insult, including IL-1r1 and COX2 (Fig. 7*D*,*E*).

Because the epilepsy-predicting change in T2 relaxation time took place in the amygdala complex, we examined in an additional cohort of animals whether cytokine expression in the amygdala correlated with T2 signal changes in the same animal. In this cohort, 7 of 11 eFSE animals had T2 relaxation times lower than 2 SDs of control (“affected”; Fig. 7*F*). Amygdala cytokine mRNA levels at 24 h after eFSE are shown in Figure 7*G*, dividing the cohort into affected and nonaffected. As apparent, a subgroup of inflammatory mediators, including IL-1r1, TLR4, and IL-6, seemed to distinguish between the groups. When amygdalar cytokine mRNA levels were plotted against T2 relaxation times, only IL-1r1 was significantly correlated (Fig. 7*H*). Comparing the correlations of hippocampal and amygdala cytokine levels and the predictive T2 signal change, IL-1r1 was common to both regions. In addition, COX2 correlated with the MRI changes in hippocampus only. A weak correlation between mRNA levels of TLR4, a target of HMGB1with MRI changes was observed (*p* = 0.15, Spearman *r* = −0.33; Fig. 7*I*). Together, these data suggest that individual differences among rats in the type and degree of inflammatory response to eFSE in at least two limbic regions implicated in epileptogenesis may underlie MRI signal changes that predict the eventual development of epilepsy in the same rats. The data support the notion that specific subsets of inflammatory molecules contribute to epileptogenesis following eFSE in individual animals.

## Discussion

The principal findings of this series of experiments are as follows: (1) rapid and coordinate upregulated expression of a subset of inflammatory molecules takes place in hippocampus and amygdala within hours after eFSE; (2) inflammatory mediator expression profiles vary significantly among individual rats experiencing eFSE, and only partial overlap exists between hippocampal and amygdala expression patterns; and (3) inflammatory profiles of individual rats correlate with MRI T2 signal changes that predict epileptogenesis.

The earliest change detected in a subgroup of immature rats experiencing eFSE was the translocation of the cellular stress-sensitive protein HMGB1 from nucleus to cytoplasm (Rovere-Querini et al., 2004). This event, taking place within minutes to hours suggests that while eFSE does not seem to kill cells, it does provoke major cellular stress in hippocampal neurons (Toth et al., 1998; Dubé et al., 2010; Choy et al., 2014). HMGB1 translocation to the cytoplasm is typically followed by release from the cell and activation of receptors on microglia and astrocytes (including TLR4) to promote the synthesis of multiple inflammatory cytokines (Rovere-Querini et al., 2004). It should be noted that a number of other activators of TLR4 receptors (e.g., degradation products of the cellular matrix and heat shock proteins) have been reported by several groups. However, cellular debris and heat shock proteins have not been found in the eFSE model (Toth et al., 1998; Bender et al., 2004; Baram et al., 2011). In addition, whereas HMGB1 translocation in adult models of epilepsy has typically been reported in astrocytes and microglia (Maroso et al., 2010), in the eFSE model we find the translocation exclusively in neurons during the first few hours (Choy et al., 2014). This cell specificity might be a result of the seizure-inciting insult, the age of the animals, or both (Baram, 2012).

Here we found that, in hippocampus, the expression of IL-1β, its receptor (IL-1r1), TNF-α, COX2, and GFAP were upregulated during the hours and days following eFSE. Most cytokines were transiently upregulated with levels resembling those in controls by 96 h after eFSE. Only COX2 remained upregulated at this time point. Looking within the amygdala (at the 24 h time point) demonstrated similar findings, with IL-6, COX2, and TNF-α expression augmented. These quantitative analyses of cytokine expression were complemented by immunohistochemical evidence for significant astrocytosis and microglia activation 24 h after eFSE, which is indicative of an active inflammatory response in the hippocampus. Thus, whereas the expression of the cytokines measured here was transient, it might well set in motion additional downstream processes, potentially via activation of NF-κB and other transcriptional regulators (Crespel et al., 2002; Aronica et al., 2010). These downstream consequences of the original wave of select inflammatory processes might contribute to the development of epilepsy.

An important question for both clinical FSE and eFSE is why some individuals go on to have epilepsy while others do not. Whereas it is difficult to test in humans, we have tried to identify factors that might contribute to epilepsy during or after eFSE. There were no discernable differences in the initial eFSE, where the seizure duration, time to onset, and intensity were similar among rats that eventually became epileptic and those that did not (Dubé et al., 2010; Choy et al., 2014). As demonstrated here, it is unlikely that the hyperthermia alone caused toxic physiological consequences because core temperatures never elevated above 41.5°C during eFSE. In addition, whereas there was no interlitter variability in reaction to the inciting event, interanimal variability was found in each litter both in the expression of inflammatory mediators and in potential seizure outcome. The studies described here suggest that an insult across genetically similar animals might result in markedly varied inflammatory profiles. These might derive from prior inflammatory or immune challenge (Riazi et al., 2010; Galic et al., 2012) or other, as yet undiscovered, reasons. The current data tempt us to speculate that the divergent inflammatory responses in rats experiencing eFSE might, in turn, lead to divergent epileptogenic outcomes.

Interestingly, while in hippocampus both COX2 and IL-1r1 levels correlated with the epilepsy-predictive MRI signal change after eFSE, in amygdala the correlation was with IL-1r1 mRNA levels only. The divergence between regions may suggest regional inflammatory differences in the limbic system that are important for epileptogenesis. The involvement of IL-1r1 in both regions is intriguing, suggesting that this specific cytokine may be instrumental in epilepsy generated by eFSE. Previous work (Dubé et al., 2005) has demonstrated that this cytokine pathway was required for the generation of eFSE. Further work (Dubé et al., 2010) suggested that IL-1β levels distinguished adult epileptic rats from those that did not develop epilepsy following eFSE. The current work provides additional support for the notion that augmentation of this cascade in a subset of rats contributes to epileptogenesis in the same rats. Notably, blocking the interleukin-1 cascade in epileptic animals has anticonvulsive effects, but the role of this cascade in epileptogenesis is presently unclear (Maroso et al., 2011; Noe et al., 2013). This role might vary among models because of differences in animal ages and other parameters.

In summary, these correlative studies demonstrate a potential relationship between selective augmentation of subsets of inflammatory mediators after eFSE and epilepsy. Blocking inflammation as a whole (Holtman et al., 2014) has proven unhelpful for aborting epilepsy, as have a number of studies targeting specific pathways in the inflammatory molecular network (Holtman et al., 2009; Vezzani et al., 2011; Noe et al., 2013). Indeed, rather than being pathogenic, it is possible that some observed inflammatory responses may be neuroprotective against the epileptogenic process, as has been described for other disorders (Schwartz and Kipnis, 2005). Future studies will focus on dissecting out which of the inflammatory cytokines and mediators described here are epileptogenic and which might be important for restoring normal neuronal function.

## Synthesis

The decision was a result of the Reviewing Editor Quentin Pittman and the peer reviewers coming together and discussing their recommendations until a consensus was reached. A fact-based synthesis statement explaining their decision and outlining what is needed to prepare a revision is listed below. The following reviewers agreed to reveal their identity: Alfonso Represa

The manuscript is well-written and provides interesting data. The inter-individual variations of inflammatory response is particularly interesting and most likely highly relevant. However, the reviewers think that it is still a bit preliminary and some points deserve clarification or more data:

1. Both reviewers are wanting to have a bit more evidence for a causative relation,. In its current form , the manuscript looks at inflammatory changes in the hippocampus , but not in the amygdala; because we don't know what a change in T2 relaxation time really means (even in humans) it is thought to be very important to discuss any causative link between this biomarker and the occurrence of epilepsy. The reviewers suggest 2 avenues: 1) look at the cytokine levels in the amygdala where the T2 changes are found and 2) T2 relaxation time was found to correlate with COX2 mRNA and IL-1r1 mRNA levels. To confirm their potentiality as biomarkers it would be important to evaluate T2 relaxation time and correlations with inflammatory markers: i) after inflammation with no-seizures, ii) after inhibition of part of this inflammatory response (e.g. COX2 as described in the report by Oliveira MS et al. 2008), iii) after hyperthermia with no-seizures.

2. The reviewers also point out that while you have investigated essentially the expression levels of mRNAs for major inflammatory messengers, whether mRNA levels directly reflect protein levels has been highly challenged over the last decade and it remains important to complete the data by semi-quantitative analysis of proteins levels.

3. As mentioned by the authors, HMGB1 suggest major cellular stress. The manuscript needs to include a concomitant assessment of cell injury at the same study time points (H&amp;E staining or fluorojade staining). If this was assessed in the early description of this model, this is not clearly assessed for longer duration of hyperthermia exposure/ seizure duration. This is also required regarding the inter-individual variability This point would further reinforce the role of inflammation by itself if no cell injury is reported.

Other points/questions that should be addressed include:

4. Did authors include in the analysis animals with core temperature higher than 41.5? What was the mean core temperature of explored animals? Did core temperature evolve during the seizure period?

5. How many litters were used? How much of the variability was a litter effect? Also please provide details like- were pregnant dams transported ( a huge source of variable stress ( eg Moriyama et al 2013)? Please ensure that all figures indicate `n`.

6. It is also interesting in Fig 6 C the Il-6 levels were higher in èxpected epilepsy, yet it does not appear in the correlative graphs shown in D-G. Is there a reason for this? Was the expected correlation not present?

7. Authors used ANOVA (figure 4) for statistical analysis of non-Gaussian data distribution ; a non-parametric test must be used instead (e.g. Kruskal-Wallis).

COMMENTS-REVIEWER 1

This work aims to identify new biomarkers predictive for the development of temporal lobe epilepsy (TLE) after long febrile seizures in childhood. The report investigates the issue in an animal model of hyperthermia described by Dubé et al (2010) in which only a subset of animals develops an epileptic condition and compared the level of expression of mRNAs encoding some inflammatory molecules with the reduction of T2 relaxation times in the basolateral amygdala (high-field MRI) within hours after the febrile status epilepticus, a protocol which accordingly to Choi et al (20014) would be predictive for epilepsy occurrence.

The report is interesting but I think that it is still a bit preliminary and some points deserve clarification:

• Authors investigated essentially the expression levels of mRNAs for major inflammatory messengers. Whether mRNA levels directly reflect protein levels has been highly challenged over the last decade and it remains important to complete the data by semi-quantitative analysis of proteins levels.

• Inflammatory responses were investigated in the hippocampal complex, however MRI analysis concentrate in the amygdala. I think that correlative analysis of MRI changes and inflammatory responses must be performed on the same structure and I therefore suggest to provide in addition to data presented here both T2 relaxation times in the hippocampus and inflammatory changes in the amygdala.

• Did all experimental animals with hyperthermia display a status epilepticus? Did authors include in the analysis animals with core temperature higher than 41.5? What was the mean core temperature of explored animals? Did core temperature evolve during the seizure period?

• T2 relaxation time was found to correlate with COX2 mRNA and IL-1r1 mRNA levels. This observation requires further investigations to reinforce their relationship and confirm their potentiality as biomarkers. I think it would be important to evaluate T2 relaxation time and correlations with inflammatory markers: i) after inflammation with no-seizures, ii) after inhibition of part of this inflammatory response (e.g. COX2 as described in the report by Oliveira MS et al. 2008), iii) after hyperthermia with no-seizures. It is likely that animals so treated will not develop a TLE, thus providing a good mean to validate the selectivity of these inflammatory parameters and T2 relaxation time as biomarkers.

• Authors used ANOVA (figure 4) for statistical analysis of non-Gaussian data distribution ; a non-parametric test must be used instead (e.g. Kruskal-Wallis)

COMMENTS REVIEWER 2

This manuscript report the changes in inflammation induced by status epilepticus induced by hyperthermia. The manuscript is well-written and provides interesting data. The inter-individual variations of inflammatory response is particularly interesting and most likely highly relevant.

Few points need to be addressed:

1. As mentioned by the authors, HMGB1 suggest major cellular stress. The manuscript needs to include a concomitant assessment of cell injury at the same study time points (H&amp;E staining or fluorojade staining). If this was assess in the early description of this model, this is not clearly assess for longer duration of hyperthermia exposure/ seizure duration. This is also required regarding the inter-individual variability This point would further reinforce the role of inflammation by itself if no cell injury is reported.

2. Why the authors did not explore the inflammatory response in the amygdala? They should at least provide some data at 2-4h time point when a decreased MRI T2 relaxation time has been described.

## References

[B1] Annegers JF, Hauser WA, Shirts SB, Kurland LT (1987) Factors prognostic of unprovoked seizures after febrile convulsions. N Engl J Med 316:493–498. 10.1056/NEJM198702263160901 3807992

[B2] Aronica E, Fluiter K, Iyer A, Zurolo E, Vreijlin E, van Vliet EA, Baayen JC, Gorter JA (2010) Expression pattern of miR-146a, an inflammation-associated microRNA, in experimental and human temporal lobe epilepsy. Eur J Neurosci 31:1100-1107. 10.1111/j.1460-9568.2010.07122.x 20214679

[B3] Baram TZ (2012) The brain, seizures and epilepsy throughout life: understanding a moving target. Epilepsy Curr 12:7-12. 10.5698/1535-7511-12.4s.7 23476117 PMC3588148

[B4] Baram TZ, Gerth A, Schultz L (1997) Febrile seizures: an appropriate-aged model suitable for long-term studies. Dev Brain Res 98:265-270. 9051269 10.1016/s0165-3806(96)00190-3 PMC3404508

[B5] Baram TZ, Jensen FE, Brooks-Kayal A (2011) Does acquired epileptogenesis in the immature brain require neuronal death? Epilepsy Curr 11:21-26. 21461261 10.5698/1535-7511-11.1.21 PMC3063568

[B6] Bender RA, Dubé C, Baram TZ (2004) Febrile seizures and mechanisms of epileptogenesis: insights from an animal model. Adv Exp Med Biol 548:213-225. 15250596 10.1007/978-1-4757-6376-8_15 PMC3086822

[B7] Berg AT, Shinnar S (1996) Complex febrile seizures. Epilepsia 37:126-133. 8635422 10.1111/j.1528-1157.1996.tb00003.x

[B8] Berg AT, Shinnar S, Darefsky AS, Holford TR, Shapiro ED, Salomon ME, Crain EF, Hauser AW (1997) Predictors of recurrent febrile seizures: a prospective cohort study. Arch Pediatr Adolesc Med 151:371-378. 9111436 10.1001/archpedi.1997.02170410045006

[B52] Bianchi ME, Manfredi AA (2007) HIgh mobility group box 1 (HMGB1) protein at the crossroads between innate and adaptive immunity. Immunological reviews 200(1): 35–46. 10.1111/j.1600-065X.2007.00574.x 17979838

[B9] Cendes F, Andermann F, Dubeau F, Gloor P, Evans A, Jones-Gotman M, Olivier A, Andermann E, Robitaille Y, Lopes-Cendes I (1993) Early childhood prolonged febrile convulsions, atrophy and sclerosis of mesial structures, and temporal lobe epilepsy: an MRI volumetric study. Neurology 43:1083–1087. 8170546 10.1212/wnl.43.6.1083

[B10] Choy, M, Dubé, CM, Patterson KP, Barnes SR, Maras P, Blood A B, Hasso An, Obenaus A, Baram TZ (2014) A novel, noninvasive, predictive epilepsy biomarker with clinical potential. J Neurosci 34:8672–8684. 10.1523/JNEUROSCI.4806-13.2014 24966369 PMC4069350

[B11] Crespel A, Coubes P, Rousset MC, Brana C, Rougier A, Rondouin G, Bockaert J, Baldy-Moulinier M, Lerner-Natoli M (2002) Inflammatory reactions in human medial temporal lobe epilepsy with hippocampal sclerosis. Brain Res 952:159-169. 12376176 10.1016/s0006-8993(02)03050-0

[B12] Dedeurwerdere S, Friedman A, Fabene PF, Mazarati A, Murashima YL, Vezzani A, Baram TZ (2012) Finding a better drug for epilepsy: antiinflammatory targets. Epilepsia 53:1113-1116. 22691043 10.1111/j.1528-1167.2012.03520.x PMC3389561

[B13] De Simoni MG, Perego C, Ravizza T, Moneta D, Conti M, Marchesi F, De Luigi, A, Garattini S, Vezzani A (2000) Inflammatory cytokines and related genes are induced in the rat hippocampus by limbic status epilepticus. Eur J Neurosci 12:2623-2633. 10947836 10.1046/j.1460-9568.2000.00140.x

[B14] Dubé, C, Chen K, Eghbal-Ahmadi M, Brunson K, Soltesz I, Baram TZ (2000) Prolonged febrile seizures in the immature rat model enhance hippocampal excitability long term. Ann Neurol 47:336-344. 10716253 PMC3139468

[B15] Dubé C, Yu H, Nalcioglu O, Baram TZ (2004) Serial MRI after experimental febrile seizures: altered T2 signal without neuronal death. Ann Neurol 56:709–714. 10.1002/ana.20266 15389889 PMC3084032

[B16] Dubé C, Vezzani A, Behrens M, Bartfai T, Baram TZ (2005) Interleukin-1beta contributes to the generation of experimental febrile seizures. Ann Neurol 57:152–155. 10.1002/ana.20358 15622539 PMC2909879

[B17] Dubé CM, Brewster AL, Richichi C, Zha Q, Baram TZ (2007) Fever, febrile seizures and epilepsy. Trends Neurosci 30:490-496. 10.1016/j.tins.2007.07.006 17897728 PMC2766556

[B18] Dubé CM, Zhou JL, Hamamura M, Zhao Q, Ring A, Abrahams J, McIntyre K, Nalcioglu O, Shatskih T, Baram TZ, Holmes GL (2009a) Cognitive dysfunction after experimental febrile seizures. Exp Neurol 215:167-177. 10.1016/j.expneurol.2008.10.003 19000675 PMC2649663

[B19] Dubé CM, Brewster AL, Baram TZ (2009b) Febrile seizures: mechanisms and relationship to epilepsy. Brain Dev 31:366–371. 10.1016/j.braindev.2008.11.010 19232478 PMC2698702

[B20] Dubé CM, Ravizza T, Hamamura M, Zha Q, Keebaugh A, Fok K, Andres AL, Nalcioglu O, Obenaus A, Vezzani A, Baram TZ (2010) Epileptogenesis provoked by prolonged experimental febrile seizures: mechanisms and biomarkers. J Neurosci 30:7484–7494. 10.1523/JNEUROSCI.0551-10.2010 20519523 PMC2906240

[B21] Eskilsson A, Mirrasekhian E, Dufour S (2014) Immune-induced fever is mediated by IL-6 receptors on brain endothelial cells coupled to STAT3-dependent induction of brain endothelial prostaglandin synthesis. J Neurosci 34:15957-15961. 10.1523/JNEUROSCI.3520-14.2014 25429137 PMC6608482

[B22] French JA, Williamson PD, Thadani VM, Darcey TM, Mattson RH, Spencer SS, Spencer DD (1993) Characteristics of medial temporal lobe epilepsy: I. Results of history and physical examination. Ann Neurol 34:774-780. 10.1002/ana.410340604 8250525

[B23] Galic MA, Riazi K, Pittman QJ (2012) Cytokines and brain excitability. Front Neuroendocrinol 33:116-125. 10.1016/j.yfrne.2011.12.002 22214786 PMC3547977

[B24] Heida JG, Pittman QJ (2005) Causal links between brain cytokines and experimental febrile convulsions in the rat. Epilepsia 46:1906-1913. 10.1111/j.1528-1167.2005.00294.x 16393156

[B25] Heida JG, Boissé L, Pittman QJ (2004) Lipopolysaccharide- induced febrile convulsions in the rat: short-term sequelae. Epilepsia 45:1317-1329. 10.1111/j.0013-9580.2004.13704.x 15509232

[B26] Heida JG, Teskey GC, Pittman QJ (2005) Febrile convulsions induced by the combination of lipopolysaccharide and low-dose kainic acid enhance seizure susceptibility, not epileptogenesis, in rats. Epilepsia 46:1898-1905. 10.1111/j.1528-1167.2005.00286.x 16393155

[B27] Heida JG, Moshé SL, Pittman QJ (2009) The role of interleukin-1β in febrile seizures. Brain Dev 388-393. 10.1016/j.braindev.2008.11.013 19217733 PMC2699664

[B28] Hellier JL, Patrylo PR, Buckmaster PS, Dudek FE (1998) Recurrent spontaneous motor seizures after repeated low-dose systemic treatment with kainate: assessment of a rat model of temporal lobe epilepsy. Epilepsy Res 31:73-84. 10.1016/S0920-1211(98)00017-5 9696302

[B29] Holtman L, van Vliet EA, van Schaik R, Queiroz CM, Aronica E, Gorter JA (2009) Effects of SC58236, a selective COX-2 inhibitor, on epileptogenesis and spontaneous seizures in a rat model for temporal lobe epilepsy. Epilepsy Res 84:56-66. 10.1016/j.eplepsyres.2008.12.006 19186029

[B30] Holtman L, van Vliet EA, Edelbroek PM, Aronica E, Gorter JA (2010) Cox-2 inhibition can lead to adverse effects in a rat model for temporal lobe epilepsy. Epilepsy Res 91:49-56. 10.1016/j.eplepsyres.2010.06.011 20643531

[B31] Holtman L, van Vliet EA, Appeldoorn C, Gaillard PJ, de Boer M, Dorland R, Wadman WJ, Gorter JA (2014) Glutathione pegylated liposomal methylprednisolone administration after the early phase of status epilepticus did not modify epileptogenesis in the rat. Epilepsy Res 108:396-404. 10.1016/j.eplepsyres.2014.01.010 24556423

[B32] Jansen JFA, Lemmens EMP, Strijkers GJ, Prompers JJ, Schijns OEMG, Kooi ME, Beuls EAM, Nicolay K, Backes WH, Hoogland G (2008) Short- and long-term limbic abnormalities after experimental febrile seizures. Neurobiol Dis 32:293-301. 10.1016/j.nbd.2008.07.010 18707002

[B33] Lenz KM, Nugent BM, Haliyur R, McCarthy MM (2013) Microglia are essential to masculinization of brain and behavior. J Neurosci 33:2761–2772. 10.1523/JNEUROSCI.1268-12.2013 23407936 PMC3727162

[B34] Lewis DV, Shinnar S, Hesdorffer DC (2014) Hippocampal sclerosis after febrile status epilepticus: the FEBSTAT study. Ann Neurol 75:178-185. 10.1002/ana.24081 24318290 PMC3980500

[B35] Luheshi GN, Stefferl A, Turnbull AV (1997) Febrile response to tissue inflammation involves both peripheral and brain IL-1 and TNF-alpha in the rat. Am J Physiol 272:R862–R868. 9087648 10.1152/ajpregu.1997.272.3.R862

[B36] Maroso M, Balosso S, Ravizza T, Liu J, Aronica, E, Iyer A. M, Rossetti, C, Molteni M, Casalgrandi M, Manfredi AA, Bianchi ME, Vezzani A (2010) Toll-like receptor 4 and high-mobility group box-1 are involved in ictogenesis and can be targeted to reduce seizures. Nat Med 16:413–419. 10.1038/nm.2127 20348922

[B37] Maroso M, Balosso S, Ravizza T, Iori V, Wright CI, French J, Vezzani A (2011) Interleukin-1 biosynthesis inhibition reduces acute seizures and drug resistant chronic epileptic activity in mice. Neurotherapeutics 8:304-315. 10.1007/s13311-011-0039-z 21431948 PMC3101825

[B38] Noe FM, Polascheck N, Frigerio F, Bankstahl M, Ravizza T, Marchini S, Beltrame L, Reschke Banderó C, Löscher W, Vezzani A (2013) Pharmacological blockade of IL-1β/IL-1 receptor type 1 axis during epileptogenesis provides neuroprotection in two rat models of temporal lobe epilepsy. Neurobiol Dis 59:183-193. 10.1016/j.nbd.2013.07.015 23938763

[B39] Ollikainen J, Honkaniemi J, Palmio J, Peltola J (2004) Regulation of IL-6 system in cerebrospinal fluid and serum compartments by seizures: the effect of seizure type and duration. J Neuroimmunol 152:121-125. 10.1016/j.jneuroim.2004.01.024 15223244

[B40] Patterson KP, Baram TZ, Shinnar S (2014) Origins of temporal lobe epilepsy: febrile seizures and febrile status epilepticus. Neurotherapeutics 11:242–250. 10.1007/s13311-014-0263-4 24604424 PMC3996115

[B41] Pouliot WA, Dudek FE, Dingledine R (2013) Inhibition of the prostaglandin receptor EP2 following status epilepticus reduces delayed mortality and brain inflammation. Proc Natl Acad Sci U S A 110:3591-3596. 23401547 10.1073/pnas.1218498110 PMC3587237

[B42] Ravizza T, Gagliardi B, Francesco N, Boer K, Aronica E, Vezzani A (2008) Innate and adaptive immunity during epileptogenesis and spontaneous seizures: evidence from experimental models and human temporal lobe epilepsy. Neurobiol Dis 29:142-160. 10.1016/j.nbd.2007.08.012 17931873

[B43] Riazi K, Galic MA, Pittman QJ (2010) Contributions of peripheral inflammation to seizure susceptibility: cytokines and brain excitability. Epilepsy Res 89:34-42. 10.1016/j.eplepsyres.2009.09.004 19804959

[B44] Rovere-Querini P, Capobianco A, Scaffidi P, Valentinis B, Catalanotti F, Giazzon M, Dumitriu IE, Müller S, Iannacone M, Traversari C, Bianchi ME, Manfredi AA (2004) HMGB1 is an endogenous immune adjuvant released by necrotic cells. EMBO Rep 5:825-830. 10.1038/sj.embor.7400205 15272298 PMC1299116

[B45] Schwartz M, Kipnis J (2005) Protective autoimmunity and neuroprotection in inflammatory and noninflammatory neurodegenerative diseases. J Neurol Sci 233:163-166. 10.1016/j.jns.2005.03.014 15949502

[B46] Scott RC, Gadian DG, King MD, Chong WK, Cox TC (2002). Magnetic resonance imaging findings within 5 days of status epilepticus in childhood. Brain 125:1951-1959. 12183341 10.1093/brain/awf202

[B47] Seinfeld S, Shinnar S, Sun S, Hesdorffer DC, Deng X, Shinnar RC, O’Hara K, Nordli DR, Frank LM, Gallentine, W, Moshé SL, Pellock JM (2014) Emergency management of febrile status epilepticus: results of the FEBSTAT study. Epilepsia 55*:* 388–395. 10.1111/epi.12526 24502379 PMC3959260

[B48] Toth Z, Yan XX, Haftoglou, Ribak CE, Baram TZ (1998) Seizure-induced neuronal injury: vulnerability to febrile seizures in an immature rat model. J Neurosci 18:4285-4294. [Mismatch] 9592105 10.1523/JNEUROSCI.18-11-04285.1998 PMC3387924

[B49] VanLandingham KE, Heinz RE, Cavazos JE, Lewis DV (1998). Magnetic resonance imaging evidence of hippocampal injury after prolonged focal febrile convulsions. Ann Neurol 43:413–426. 10.1002/ana.410430403 9546321

[B50] Vezzani A, Balosso S, Ravizza T (2008) The role of cytokines in the pathophysiology of epilepsy. Brain 22:797-803. 10.1016/j.bbi.2008.03.009 18495419

[B51] Vezzani A, French J, Bartfai T, Baram TZ (2011) The role of inflammation in epilepsy. Nat Rev Neurol 7:31-40. 10.1038/nrneurol.2010.178 21135885 PMC3378051

